# Supplementary material for: Tead1a Initiates Transcriptional Priming Through the TEAD1a/YAP‐Notch1‐Spi1/Cebpα Axis to Promote Neutrophil Fate
Source: Adv Sci (Weinh). 2025 Aug 28;12(41):e05441. doi: 10.1002/advs.202505441 (PMC12591127; doi:10.1002/advs.202505441)
Supplement: Supplementary file 4 — Supplementary Dataset [file ADVS-12-e05441-s004.zip › 3-5.zip!/3-5/02-5.cluster/cluster14/Enrich/ego_all.pdf]

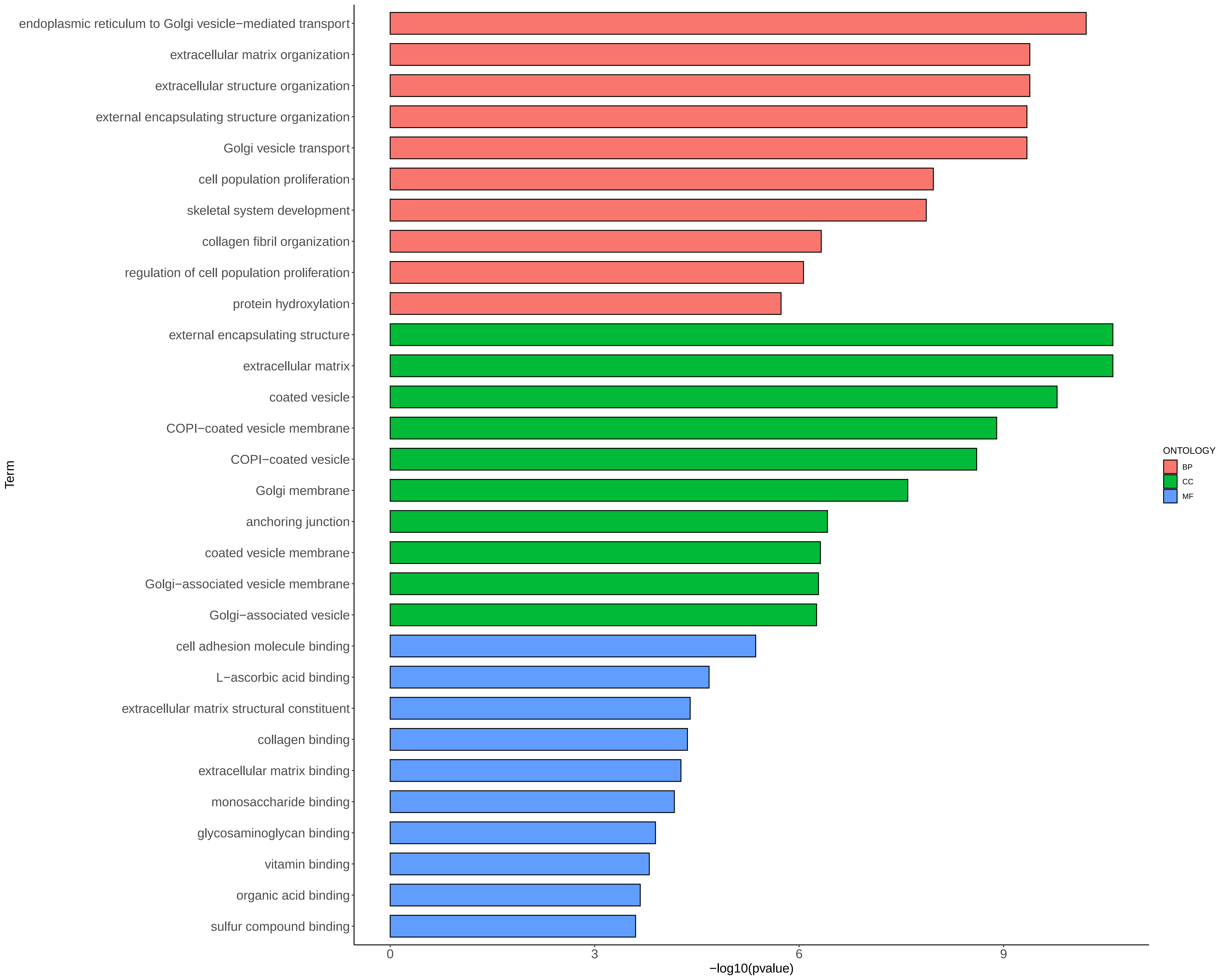

endoplasmic reticulum to Golgi vesicle−mediated transport
extracellular matrix organization
extracellular structure organization
external encapsulating structure organization
Golgi vesicle transport
cell population proliferation
skeletal system development
collagen fibril organization
regulation of cell population proliferation
protein hydroxylation
external encapsulating structure
extracellular matrix
coated vesicle
COPI−coated vesicle membrane
COPI−coated vesicle
Golgi membrane
anchoring junction
coated vesicle membrane
Golgi−associated vesicle membrane
Golgi−associated vesicle
cell adhesion molecule binding
L−ascorbic acid binding
extracellular matrix structural constituent
collagen binding
extracellular matrix binding
monosaccharide binding
glycosaminoglycan binding
vitamin binding
organic acid binding
sulfur compound binding
Term
0
3
6
9
−log10(pvalue)
ONTOLOGY
BP
CC
MF
